# A reversible feedback mechanism regulating mitochondrial heme synthesis

**DOI:** 10.1016/j.jbc.2025.111089

**Published:** 2025-12-22

**Authors:** Iva Chitrakar, Alexis B. Roberson, Pedro H. Ayres-Galhardo, Breann L. Brown

**Affiliations:** 1 Department of Biochemistry, Vanderbilt University School of Medicine, Nashville, Tennessee, USA; 2 Department of Chemistry, Fisk University, Nashville, Tennessee, USA; 3 Center for Structural Biology, Vanderbilt University School of Medicine, Nashville, Tennessee, USA

**Keywords:** heme, enzyme inhibition, erythropoiesis, aminolevulinic acid, pyridoxal 5-phosphate, heme regulatory motif, protein structure and function, enzymology

## Abstract

Proper heme biosynthesis is essential for numerous cellular functions across nearly all life forms. In humans, dysregulated heme metabolism is linked to multiple blood diseases, neurodegeneration, cardiovascular disease, and metabolic disorders. Erythroid heme production begins with the rate-limiting enzyme aminolevulinic acid synthase 2 (ALAS2) in the mitochondrion. Although prior studies discuss the regulation of ALAS2 in the cytoplasm, its modulation as a mature mitochondrial matrix enzyme remains poorly understood. We report that heme binds mature human ALAS2 with high affinity, acting as a reversible mixed inhibitor that reduces enzymatic activity. Structural modeling supports the hypothesis that two flexible regions of ALAS2 interact with heme, locking the enzyme in an inactive conformation and occluding the active site. Our work reveals a negative feedback mechanism for heme synthesis, offering insights into the spatial regulation of ALAS2 and the maturation of the essential heme cofactor.

Our knowledge of cellular metabolism continues to expand with our understanding of the role of metabolites as cell signaling effectors (reviewed in (1)). Consequently, metabolic processes, which form the foundation of organismal biology, are studied in a new context to reveal critical insights into chemical and cellular signaling. Heme metabolism is central to multiple biological processes, as it is a vital cofactor supporting oxygen transport, transcriptional regulation, and drug detoxification, among others (2, 3, 4). Heme production must be tightly controlled due to the toxicity associated with heme deficiency or excessive amounts of porphyrin precursors, which produce reactive oxygen species and free radicals (5, 6, 7, 8).

The enzyme that catalyzes the rate-limiting step for heme biosynthesis in many organisms is aminolevulinic acid synthase (ALAS) (9, 10), a PLP-dependent homodimer enzyme that mediates the condensation of glycine and succinyl-CoA to produce aminolevulinic acid (11, 12). Humans contain two ALAS isoforms, ALAS1 and ALAS2 (13, 14, 15). ALAS1 is a ubiquitously expressed housekeeping enzyme (13, 14), and ALAS2 is the erythroid-specific isoform that governs the synthesis of 85 to 90% of the total body heme pool (5, 16). The high demand for heme to produce hemoglobin implies that erythroid heme synthesis is not tightly regulated. However, the fact that multiple blood disorders arise from dysfunctional heme biosynthesis indicates otherwise. Current clinical reports have identified over 95 human *ALAS2* mutations that result in the loss-of-function disease X-linked sideroblastic anemia (XLSA) (17, 18, 19). or the gain-of-function disease X-linked protoporphyria (XLPP) (20, 21). Compelling new evidence reveals that *ALAS2* is also expressed in nonerythroid cells (22), underscoring our critical need to understand ALAS2 enzyme regulation for metabolic processes beyond erythropoiesis.

Previous research primarily focused on the nuclear and cytosolic regulation of *ALAS* expression and trafficking (23, 24, 25, 26, 27, 28, 29, 30, 31, 32, 33). For example, heme binds directly to the ALAS mitochondrial targeting sequence, preventing protein translocation into the matrix (23, 24, 34, 35, 36). However, little is known about the enzymatic modulation of ALAS within the mitochondrial matrix, which is the cellular compartment where heme biosynthesis is initiated and terminated. Human ALAS paralogs contain multiple putative heme-binding motifs in the mature enzyme, which lacks the mitochondrial targeting sequence (reviewed in (37)). Various reports established that the interaction between heme and mitochondrial ALAS1 results in either LONP1- or CLPXP-mediated degradation (27, 38, 39). Furthermore, ALAS homologs from various α-proteobacteria are reported to be inactivated by heme binding (40, 41, 42). Whether human ALAS2 is subject to negative regulation by heme in mitochondria remains to be fully explored. Due to the complex impacts of heme variation on effective erythropoiesis, gene transcription, and several other cellular signaling pathways, it is essential to establish *in vitro* principles of enzyme regulation that form the foundation of interpreting physiological outcomes.

Here, we identify a reversible mechanism by which heme inhibits its synthesis by affecting mature mitochondrial ALAS2 activity, challenging the idea that erythroid heme synthesis is an all-or-nothing process. This mechanism responds to heme stress by quickly inactivating ALAS2, thereby reducing heme production. Along with a revised understanding of ALAS2 tissue expression (22), our study describes a new form of negative feedback in heme biosynthesis that regulates erythropoiesis and possibly the maturation of hemoproteins, thus controlling numerous biological functions.

## Results

### The human ALAS2 sequence contains multiple heme-binding motifs

One challenge to identifying transient or regulatory heme-protein interactions is the diversity of interaction motifs and amino acid ligands. Although many heme-binding proteins use histidine residues as the axial ligand to coordinate the heme iron, other examples employ cysteine, methionine, tyrosine, or lysine residues (43). The lower abundance of nonhistidine motifs makes predicting these sites less accurate than other canonical motifs (44, 45, 46). However, certain parameters have been identified to aid in prediction.

**Figure 1:**
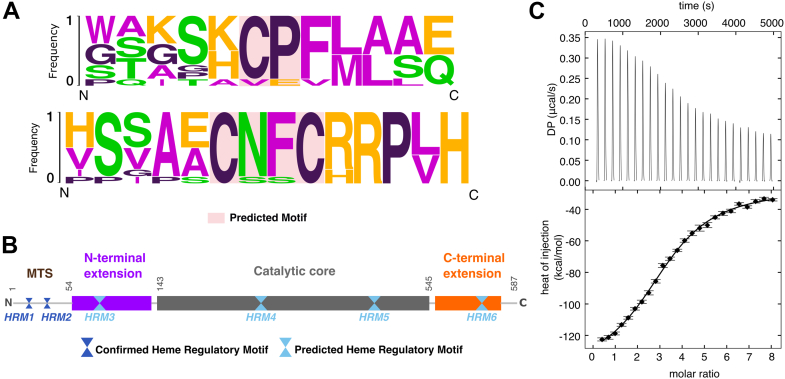
**Conserved heme regulatory motifs in ALAS.***A*, consensus sequence logo generated based on the heme regulatory motifs (HRMs) conserved across multiple ALAS homologs. Conserved motifs (*pink box*) include CP dipeptide and CXXC. *B*, domain map of human ALAS2 highlighting the experimentally confirmed HRMs in the mitochondrial targeting sequence (MTS) and predicted HRMs in *dark* and *light blue*, respectively. The ALAS N-terminal extension is colored *purple* and the C-terminal extension is colored *orange*. The motifs according to human ALAS2 residue numbers are HRM1: ^11^CP^12^; HRM2: ^38^CP^39^; HRM3: ^70^CP^71^; HRM4: ^338^CP^339^; HRM5: ^471^CP^472^; and HRM6: ^555^CXXC^558^. *C*, representative ITC thermograph (*top*) and binding isotherm (*bottom*) for the binding between hemin and WT human ALAS2. ALAS, aminolevulinic acid synthase; CP, cysteine-proline.

Previous studies identified a heme regulatory motif (HRM) that consists of a core cysteine-proline (CP) di-peptide in human ALAS1, with cysteine functioning as the axial ligand to the heme iron (23). A multiple sequence alignment of vertebrate ALAS enzymes revealed the presence of five CP motifs conserved in both isoforms across homologs (Fig. 1*A*, Fig. S1). The first two N-terminal HRMs reside in the mitochondrial targeting sequence, and heme binding to these sites negatively regulates ALAS mitochondrial import (23, 24). The remaining three motifs are dispersed throughout the N-terminal extension and catalytic core of ALAS (Fig. 1*B*).

In addition to the CP motif, a variation of a separate conserved heme-binding motif commonly found in cytochromes was also identified. This canonical CXXCH sequence, where X represents any amino acid, is found in the human ALAS2 C-terminal domain as CXXCR. Interestingly, the mouse homolog contains the classical CXXCH motif in the same position. In contrast, many other vertebrate ALAS enzymes retain the Arg residue in the terminal position (Fig. 1*A*). Collectively, ALAS2 contains 2 verified and 4 predicted HRMs per protomer, designated HRM1 through HRM6 in this article.

### Human ALAS2 binds heme *in vitro*

Given the various motifs present in human ALAS2, we sought to determine whether heme regulates mature ALAS2 after it is processed in the mitochondrial matrix. Mature wildtype ALAS2 (WT, residues 54–587), which lacks the mitochondrial targeting sequence, was expressed and purified to homogeneity. Titration of WT ALAS2 into hemin (ferric chloride heme) resulted in robust binding exhibited by an exothermic binding curve (Fig. 1*C*). The ALAS2-heme dissociation constant was determined to be ∼230 nM (Table 1). The binding stoichiometry was best fit to 4:1 (monomer equivalents), corresponding to 2 heme binding sites per ALAS2 protomer. Thus, heme binds mature ALAS2 tightly and potentially at multiple sites.

**Table 1:** Isothermal titration calorimetry thermodynamic parameters

Construct	K_d_ (nM)	ΔH (kcal/mol)	ΔS (kcal/mol^a^K)	ΔG (kcal/mol)
WT	232 [170, 324]	−382 [−440, −342]	−1.25	−9.05
ΔN	589 [322, 1428]	−1006 [−1432, −810]	−3.35	−8.50
ΔC	279 [220, 360]	−544 [−604, −499]	−1.80	−8.94
Cys-mut	610 [401, 1132]	−361 [−561, −280]	−1.18	−8.48

a 95% confidence interval in brackets.

### Heme binds reversibly to inhibit ALAS2 activity

**Figure 2:**
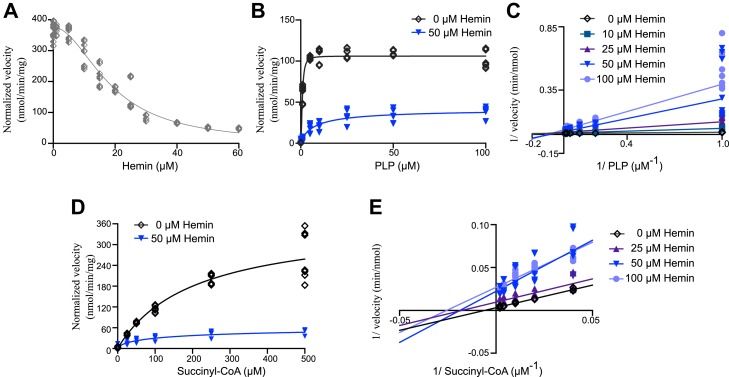
**Heme reversibly inhibits the enzymatic activity of human ALAS2.***A*, the rate of ALA product released by WT ALAS2 measured as a function of increasing hemin concentration. *B*, the rate of ALA production by WT apoALAS2 with increasing concentrations of the PLP cofactor was measured in either the absence (*black diamonds*) or the presence of 50 μM hemin (*blue triangles*). *C*, Lineweaver-Burk plot depicting the change in enzyme velocity as a function of varied PLP cofactor in the presence of increasing concentrations of the hemin inhibitor (*shades of blue*). *D*, the rate of ALA production by WT holoALAS2 with increasing concentrations of succinyl-CoA was measured in either the absence (*black diamonds*) or the presence of 50 μM hemin (*blue triangles*). *E*, Lineweaver-Burk plot depicting the change in enzyme velocity as a function of varied succinyl-CoA in the presence of increasing concentrations of the hemin inhibitor (*shades of blue*). All experiments were performed with a minimum of three biological replicates, each with three technical replicates. Data were fit using a nonlinear regression to generate an agonist dose-response curve (EC_50_) or an inhibitor dose-response curve (IC_50_). The resulting kinetic parameters (V_max_ and K_M_) were used to generate corresponding Lineweaver-Burk (double reciprocal) plots. Statistical error was determined using a one-way ANOVA.

Having established that there is robust heme binding to mature ALAS2, we sought to determine the functional impact of this interaction. The *in vitro* enzyme activity of ALAS2 was measured by monitoring the rate of ALA product release. WT ALAS2 activity decreased significantly with increasing hemin concentration, yielding an IC_50_ value of 18.7 ± 0.5 μM (Fig. 2*A*, Table 2). These studies were performed using the catalytically active holoenzyme that is covalently bound to the PLP cofactor. This covalent adduct is broken and reformed throughout the ALAS reaction cycle (Fig. S2) (47). To address the mechanism of heme inhibition, we generated a catalytically inactive form of ALAS2 by chemically cleaving the covalent bond between the endogenous PLP cofactor and a conserved active site Lys (apoALAS2). The apoenzyme is then reactivated with the addition of fresh cofactor. In the absence of heme, increasing concentrations of PLP restored enzymatic activity, with an activation constant (K_a_) of 0.72 ± 0.08 μM (Fig. 2*B*). In the presence of 50 μM heme, PLP partially rescued activity with a 7 ± 1 μM EC_50_ (Fig. 2*B*). It is possible that there is not complete restoration of maximal enzyme activity if a portion of apoALAS2 molecules contain damaged cofactor that is unable to be exchanged for exogenous PLP. Regardless, these data reveal that heme-mediated inhibition is reversible. ALAS2 apoenzyme activity was monitored with increasing amounts of PLP in the presence of various heme concentrations, up to a 100-fold excess. The resulting data revealed significant differences in maximal enzyme velocity (V_max_) and an increased PLP K_a_, indicating that heme binds as a mixed inhibitor that binds both the free and cofactor-bound enzyme (Fig. 2*C*, Table S1). We also measured the activity of purified ALAS2 holoenzyme in response to variable heme and succinyl-CoA substrate concentrations (Fig. 2*D*). Increasing amounts of succinyl-CoA still rescue heme-dependent inhibition. However, heme binds uncompetitively to the ALAS2:succinyl-CoA holoenzyme complex rather than the free enzyme, leading to decreases in both enzyme velocity (8-fold lower V_max_) and substrate affinity (4.5-fold lower K_M_, Fig. 2*E*, Table S1). Due to the sequential nature of the ALAS2 reaction, we propose that the uncompetitive inhibition results from diminished free enzyme available at this stage of the catalytic cycle. Taken together, these data establish that heme acts as an inhibitor of mature human ALAS2, binding to allosteric sites to decrease enzyme activity.

**Table 2:** ALAS2-ligand dose-response values

Construct	IC_50_ (μM)^a^	EC_50_ (μM)^b^
WT	18.7 ± 0.5	7 ± 1
ΔN	35.8 ± 0.4	4 ± 2
ΔC	31.3 ± 0.8	9 ± 1
Cys-mut	24.8 ± 0.5	10 ± 4

a IC_50_ values were determined by titrating 0.1 μM–60 μM hemin into holoALAS2_54-587_.

b EC_50_ values were determined by measuring the activity of apoALAS2 variants in the presence of 50 μM hemin (or 25 μM hemin for ΔN) with increasing PLP titration.

### Heme binding putatively favors a catalytically inactive ALAS2 conformation

**Figure 3:**
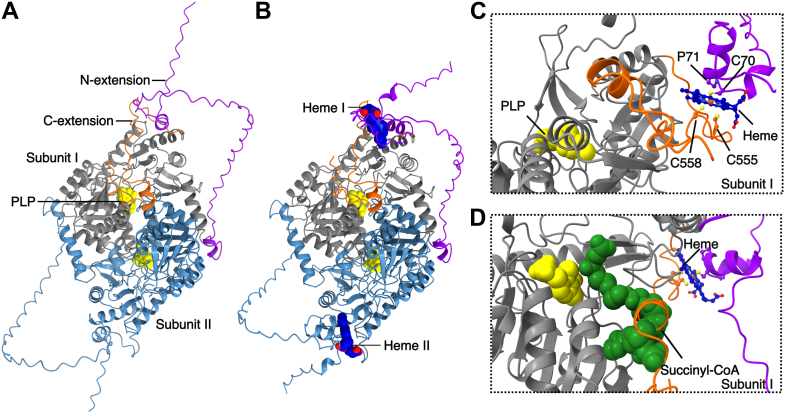
**AlphaFold3 model of the ALAS2:heme complex.** Two copies of mature ALAS2 (residues 54–587) were modeled without heme (*A*) and with two heme b molecules (*B*). The ALAS protomers are colored *gray* and *light blue*, the PLP cofactor is shown as *yellow spheres*, and the heme molecules are shown as *blue spheres*. The flexible N-terminal extension is colored *purple*, and the autoinhibitory C-terminal extension is shown in *orange*. *C*, magnified view of one heme-binding sites reveals heme coordinated between HRM3 (C70-P71) and the C-terminal HRM6 (residues 555–558) from the same subunit. *D*, addition of the succinyl-CoA substrate (*green spheres*) reveals the heme-bound model adopts an inactive conformation where the C-terminal extension sterically interferes with substrate binding. The PLP cofactor and succinyl-CoA substrate were modeled based on superposition with *Rhodobacter capsulatus* ALAS (PDB 2BWO). ALAS, aminolevulinic acid synthase; HRM, heme regulatory motif; PDB, Protein Data Bank.

The crystal structure of human ALAS2 lacks the HRM3 region and portions of the C-terminal extension due to conformational flexibility and crystallographic disorder (48). To determine how heme interacts with ALAS2 to inhibit enzyme activity, we used AlphaFold3 (AF3) to model the mature ALAS2 homodimer with and without heme (49) (Fig 3, *A* and *B*, Fig. S3). In the absence of heme, the ALAS2 dimer is largely structured in the catalytic domains, while the N- and C-terminal extensions mostly adopt a random coil conformation. Given the predicted flexibility of these regions, the AF3 prediction confidence is low, with average predicted local distance difference test (pLDDT) values of 32 and 75 for the N termini and C termini, respectively, compared to a pLDDT of 95 for the conserved catalytic domain. There are slight changes when two heme b molecules are modeled with the ALAS2 dimer, with an approximate 0.36 Å RMSD between the apo and bound structures. The most significant difference between the two models is the positioning of a long, disordered loop adjacent to a helix containing HRM3 (res 85∼126). The highest scoring model of two mature ALAS2 chains and two heme b molecules identified a potential heme binding site involving the coordination of two HRMs in the N-terminal and C-terminal extensions (Fig. 3, *B* and *C*). The predicted flexibility of these regions in the absence of heme would allow for the extensive rearrangement required to coordinate heme simultaneously (50, 51). Although the heme-binding site is predicted with lower confidence (pLDDT ∼30), all models generated by AF3 depict heme coordinated between HRM3 and HRM6 (Fig. S3). Notably, the manner of heme binding locks the C-terminal extension in a conformation that occludes the active site (Fig. 3*D*). This represents a similar inactive conformation identified in the crystal structure, indicating this orientation is energetically stable (48). The confidence of the AlphaFold model is insufficient to indicate which of the three cysteine residues present in HRM3 and HRM6 serves as the axial ligand to coordinate the heme iron center. Also, adding more than two heme molecules to the prediction led to spurious models, indicating that the remaining heme-binding sites are buried or require a more extensive conformational rearrangement that cannot be accurately modeled.

### Heme potentially binds at multiple sites to inhibit ALAS2 activity

**Figure 4:**
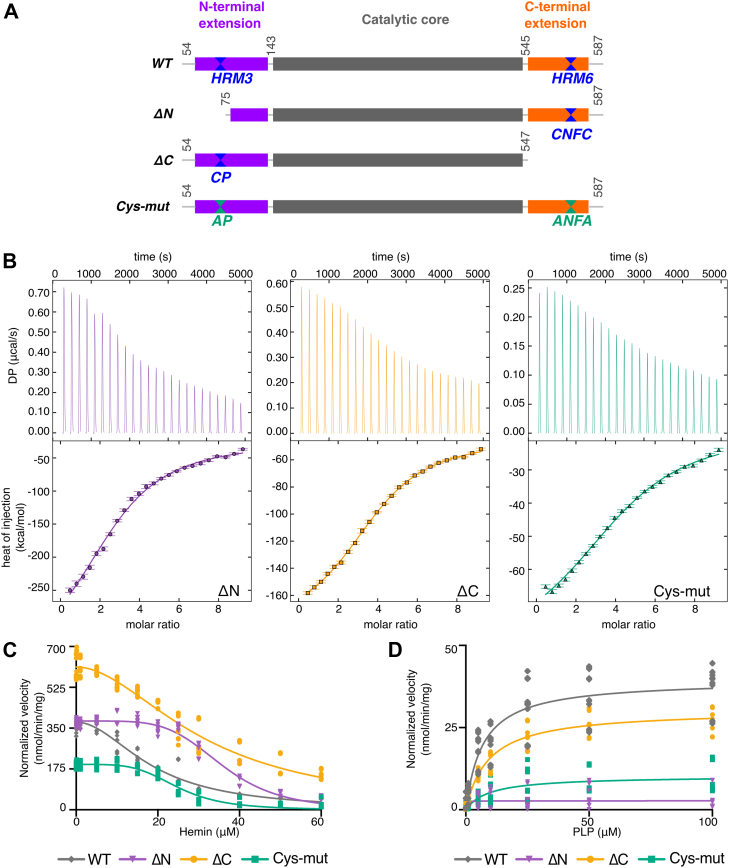
**Heme binds human ALAS2 at multiple sites.***A*, domain map of mature ALAS2 WT (colored as in Fig. 1) and HRM variants: WT (residues 54–587), ΔN (residues 75–587), ΔC (residues 54–547), and Cys-mut (C70A/C555A/C558A). *B*, representative ITC thermographs (*top*) and binding isotherms (*bottom*) for the interaction between hemin and ALAS2 ΔN (*purple*, *left*), ΔC (*orange*, *middle*), and Cys-mut (*green*, *right*). *C*, the rate of ALA release was measured as a function of increasing hemin concentrations for WT (*gray diamonds*), ΔN (*purple triangles*), ΔC (*orange circles*), and Cys-mut (*green squares*) to monitor inhibition of holoenzyme. *D*, the rate of ALA release was measured in the presence of either 50 μM hemin (WT, ΔC, and Cys-mut) or 25 μM hemin (ΔN) and increasing concentrations of PLP to determine reactivation of apoenzyme. All experiments were performed with a minimum of three biological replicates, each with three technical replicates. Data were fitted using nonlinear regression, and statistical significance was determined using a one-way ANOVA. ALAS, aminolevulinic acid synthase; HRM, heme regulatory motif; ITC, isothermal titration calorimetry.

Multiple ALAS2 constructs with various HRM mutations were generated to interrogate the accuracy of the AlphaFold model and to identify additional heme regulatory sites. These included a truncation without HRM3 (ΔN, residues 75–587), a truncation without the C-terminal CXXC motif (ΔC, residues 54–547), and a construct where all cysteines in these two motifs were mutated to alanines (Cys-mut, Fig. 4*A*). All four constructs were purified as holoenzymes and confirmed to be catalytically active. To determine whether ALAS2 binds hemin when one or more of the HRMs are compromised, isothermal titration calorimetry (ITC) was performed with each variant (Fig. 4*B*, Table 1). Titration of ΔN, ΔC, and Cys-mut into hemin resulted in an exothermic binding curve similar to WT. The WT enzyme exhibited the highest affinity for hemin, while disruption of the HRM at either terminus reduced affinity, with a lesser effect observed in the absence of HRM6. Mutation of HRMs at both termini resulted in the lowest affinity for hemin. The ΔN construct lacking HRM3 exhibited an enthalpically driven binding mechanism and decreased disorder, leading to less efficient binding. This implies that HRM3 plays a prominent role in heme binding compared to the other HRMs. However, other heme-binding sites appear to exist, as revealed by the residual heme binding to our HRM variant constructs.

Compared to WT ALAS2, the basal activity of the Cys-mut construct decreased by approximately 45%. In contrast, the truncated constructs, ΔN and ΔC, showed increases in activity of 5% and 70%, respectively (Fig. 4*C*). The significant increase in basal activity of ALAS2 ΔC is expected, as the deletion of the C-terminal extension is known to underlie the gain-of-function disorder XLPP (20). Despite the changes in maximal enzyme velocity, the addition of heme resulted in significant inhibition for all constructs (Fig. 4*C*). Enzymatic activity for all mutant constructs decreased with increasing heme concentrations but displayed higher IC_50_ values than WT (Fig. 4*C*, Table 2). Among the HRM variants, ALAS2 ΔN was the least sensitive to heme inhibition, exhibiting an IC_50_ nearly 2-fold higher than that of WT. Both ΔC and Cys-mut constructs were also inhibited by heme to a lesser extent than WT, with IC_50_ values approximately 1.6 and 1.3 times higher than WT, respectively. There were also notable differences in residual enzyme activity under saturating heme concentrations. The WT, ΔN, and Cys-mut constructs retained approximately 11 to 13% of basal activity in the presence of 100-fold excess heme. In contrast, ALAS2 ΔC retained ∼25% of basal activity under the same conditions. These results indicate that perturbation of either the N-terminal or C-terminal HRMs significantly reduces heme-mediated inhibition, but either motif is sufficient to facilitate ALAS2-heme interaction.

The reversibility of heme inhibition of the HRM variants was monitored by titrating PLP into the apoenzymes preincubated with 50 μM heme (or 25 μM heme for ΔN). In the presence of excess PLP, the apoenzyme activity was restored by ∼50% for WT and ΔC and to over 75% for Cys-mut and ΔN (Fig. 4*D*). Each ALAS2 variant exhibited EC_50_ values comparable to WT (Table 2). However, the maximal catalytic activity was restored to varying amounts as both constructs with HRM3 perturbations (ΔN and Cys-mut) displayed the lowest recovery. Additionally, measuring activity across multiple heme concentrations supported heme as an allosteric mixed inhibitor (Fig. S4). Overall, these findings provide insight into the reversible interaction between heme and ALAS2, emphasizing its role in regulating enzymatic activity through allosteric effects.

## Discussion

The heme biosynthetic enzyme ALAS2 serves as a critical node, governing the first and rate-limiting step of this essential metabolic pathway. Key differences in ALAS2, including tissue expression, protein interactions, and disease manifestation, necessitate examining this isoform separately from ALAS1. The interaction between heme and mitochondrial human ALAS2 has long been debated but lacked evidence. Here, we provide insights into the interaction between heme and the mature human ALAS2 isoform. Our investigation reveals a molecular mechanism of action in which heme acts as a potent metabolic inhibitor, effectively decreasing the enzymatic activity of ALAS2. Although labile heme concentrations are difficult to measure directly, estimates range from less than 1 μM (52) to greater than 20 μM in humans under nonpathogenic conditions (53). This upper estimate is commensurate with the IC_50_ values we report for mitochondrial ALAS2-heme inhibition, connecting our *in vitro* research to a plausible cellular state. Critically, this unique mode of inhibition is specific to the enzyme as it localizes to the mitochondrial matrix, where little is known about how ALAS2 responds to chemical cues, but where it primarily operates.

Remarkably, heme binding to ALAS2 exhibits several distinctive traits, including redundancy, reversibility, and allostery. This study supports a model by which multiple motifs act in concert to form a single heme-binding site. Our *in vitro* reconstitution uncovered a process that would otherwise be difficult to identify in cells, due to the pleiotropic effects of heme on transcriptional programs and erythropoiesis. The presence of multiple heme-binding sites within the enzyme likely serves as a fail-safe mechanism, ensuring the responsiveness of ALAS2 to heme even if one of the binding sites is compromised. For example, truncations of the ALAS2 C-terminal extension lead to the gain-of-function disease XLP (20, 21, 54), which would affect the enzyme’s ability to respond to heme stress if only one allosteric site were present. However, the presence of multiple nonequivalent heme-binding sites across the ALAS2 homodimer makes their isolation and identification difficult, as compensatory changes may occur with enzyme mutagenesis. A redundant mechanism for tuning ALAS2 activity in response to cellular heme levels confers a fitness advantage, and future studies aim to elucidate the unique allosteric properties of different HRMs.

Structural modeling and analysis support the hypothesis that heme functions as an allosteric effector by potentially inducing a conformational change in ALAS2 that prevents substrate binding and, consequently, enzymatic activity. A recent report proposed that heme-bound ALAS2 recruits the CLPXP protease *via* an adaptor protein to trigger degradation (55). Similarly, heme binding to the ALAS1 isoform also results in degradation (27, 38, 39, 56). Furthermore, our study indicates that heme binding may also influence the interactions of ALAS2 with proteins in the proposed heme synthesis metabolon, a complex of heme biosynthetic enzymes and other mitochondrial proteins that enhance metabolic efficiency (57, 58, 59). This potential modulation of the metabolon would serve as an additional layer of regulation, further influencing the overall efficiency and dynamics of heme production.

**Figure 5:**
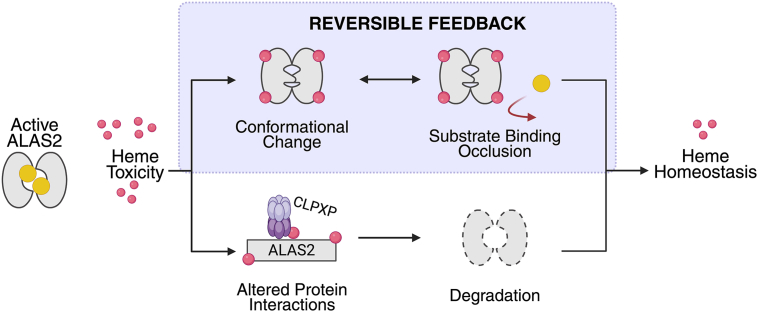
**Model of heme-mediated negative feedback inhibition of mitochondrial ALAS2.** Under basal conditions, the ALAS2 homodimer (*gray ovals*) binds PLP (*yellow sphere*) to subsequently produce heme (*red spheres*). In the case of toxic heme accumulation, heme binds to ALAS2, with potentially multiple outcomes. We discovered a mechanism where heme binding results in a conformational change that excludes substrate binding (*purple box*). In addition, heme binding may recruit mitochondrial proteases, such as CLPXP, for enzyme degradation. ALAS, aminolevulinic acid synthase.

Intriguingly, this heme-mediated inhibition discovered in our current work can coexist with a slower, irreversible means of heme-triggered ALAS2 degradation, providing the cell with a multifaceted mechanism to fine-tune heme production in response to various physiological demands (Fig. 5). Combined with previous studies of ALAS homologs in bacteria or other tissues (41, 42, 60, 61, 62, 63), our work reveals a conserved role in heme-mediated inhibition that has evolved to meet organism and tissue-specific needs. The tight control of heme biosynthesis is paramount, as heme plays a central role in a wide range of cellular processes, including oxygen transport, electron transport, and metabolic systems such as iron homeostasis and lipid metabolism (64, 65, 66, 67, 68, 69, 70). Given the pivotal role of heme in multiple processes, its dysregulation would also impact physiological functions dependent on other cofactors like FAD and cobalamin, exacerbating disease phenotypes such as anemia, neurotoxicity, oxidative stress, and metabolic dysfunction (66, 67, 68). Therefore, by supporting hemoprotein maturation and integrating with key cellular pathways, the intricate regulation of heme production is crucial for maintaining cellular homeostasis in response to environmental and metabolic demands.

## Experimental Procedures

### Protein expression and purification

All human ALAS2 constructs were cloned into a modified pET28b vector with a ULP1 protease-cleavable N-terminal hexahistidine-SUMO-tag (His_6_-SUMO). The plasmids were transformed into *Escherichia coli* BL21-Codon Plus (DE3)-RIL cells (Agilent Technologies) and grown in LB medium containing 25 μg/ml chloramphenicol and 50 μg/ml kanamycin at 37 °C and 250 rpm. Expression was induced at an *A*_600_ of 0.6 to 0.8 with 0.5 mM IPTG for 4 h at 22 °C and 250 rpm. Cultures were harvested by centrifugation, and the pelleted cells were stored at −80 °C.

Cells were resuspended in lysis buffer containing 25 mM Hepes, pH 8.0, 400 mM NaCl, 100 mM KCl, 2 mM MgCl_2_, 20 mM imidazole, 10% glycerol, 1 mM DTT, and 20 μM PLP with EDTA-free protease inhibitor (Thermo Fisher Scientific). Cells were lysed by high-pressure homogenization, and the lysate was cleared by centrifugation (at 4 °C for 30 min, 30,000*g*). The clear lysate was incubated with Ni^2+^-NTA agarose resin (Qiagen), pre-equilibrated in lysis buffer, for 1 h at 4 °C. After washing the resin with lysis buffer, the protein was eluted with lysis buffer containing 250 mM imidazole. The His-SUMO tag was cleaved overnight at 4 °C using ULP1 while dialyzing into a buffer containing 25 mM Hepes, pH 7.0, 100 mM KCl, 10% glycerol, 1 mM DTT, and 20 μM PLP. Tag-free protein was then passed through Ni^2+^-NTA resin pre-equilibrated with dialysis buffer to remove any remaining tagged protein. For holoenzyme preparation, the eluted protein was further purified with gel-filtration chromatography (HiLoad 16/600 Superdex 200 pg) equilibrated with 25 mM Hepes, pH 7, 150 mM KCl, 10% glycerol, and 0.5 mM tris(2-carboxyethyl)phosphine. Apoenzymes were prepared by overnight incubation of holoALAS proteins eluted after the second Ni^2+^-NTA purification step in stripping buffer (0.1 M potassium phosphate, pH 7.5, 10% glycerol, 1 mM DTT) with 5 mM hydroxylamine HCl, followed by a final gel filtration polishing step. The purified proteins were concentrated using a centrifugal filter (Amicon), flash-frozen in liquid nitrogen, and stored at −80 °C.

### Site-directed mutagenesis

Site-directed mutagenesis was performed on the mature ALAS2 (residues 54–587) construct to generate truncations and cysteine mutants targeting HRM3 and HRM6 (Table S2). All constructs were verified with Sanger sequencing and were expressed and purified as described above.

### Multiple sequence alignment

The amino acid sequences of human (*Homo sapiens*; P13196, P22557), bovine (*Bos taurus*; A6QLI6, Q3ZC31) rat (*Rattus norvegicus*; P13195, Q63147), mouse (*Mus musculus*; Q8VC19, P08680), chicken (*Gallus gallus*: P07997, P18080), and zebrafish (*Danio rerio*; Q9YHT4, Q7T2F0) ALAS1 and ALAS2 were retrieved from the UniProt database. The multiple sequence alignment was performed using Clustal Omega 1.2.4 with default parameters to identify conserved regions among the sequences. HRM 3 and 6, along with residues flanking the motifs, were subsequently used to generate a sequence frequency plot using WebLogo 3.7.4. The alignment of all sequences was visualized using ESPript 3.0.24.

### Isothermal titration calorimetry

Binding curves of the hemin-protein interaction were determined using Nano-ITC instrument (TA instruments) at 25 °C. Hemin was dissolved in 1 M NaOH and autoclaved to prepare a stock solution of 746 μM, ε_385nm_ = 58,440 M^−1^ cm^−1^. The stock solution was diluted to 1.5 μM in titration buffer containing 25 mM Hepes, pH 7, 150 mM KCl, and 0.5 mM TCEP. Protein samples were dialyzed into the titration buffer and diluted to a final concentration of 40 μM. Fixed volumes of protein were injected at 200-s intervals into the cell containing hemin. An A + B hetero-association model was used to determine the thermodynamic parameters for each variant. Data were integrated and analyzed using NITPIC and SEDPHAT and visualized using GUSSI (71, 72, 73).

### Enzyme activity assays

A discontinuous colorimetric activity assay adapted from Shoolingin-Jordan et al. and Bailey et al. was used to measure the enzyme activity (48, 74). The reaction was initiated by adding 100 nM purified enzyme to 50 mM potassium phosphate, pH 7.0, 10 mM MgCl_2_, 1 mM DTT, 100 mM glycine, and 300 μM succinyl-CoA (175 μl total). The reaction was incubated at 37 °C for 15 min and terminated with 100 μl of cold 10% trichloroacetic acid. The precipitated protein was removed by centrifugation for 5 min at 13,000*g,* and 240 μl of the supernatant was mixed with 240 μl of 1 M sodium acetate, pH 4.6. To derivatize ALA, 20 μl of acetylacetone was added to the mixture and boiled at 100 °C for 10 min. After the sample was cooled for 20 min, 100 μl aliquots were transferred into a clear 96-well plate in triplicate. An equal volume of Ehrlich’s reagent (p-Dimethylaminobenzaldehyde, DMAB) was added to each well, and the absorbance was monitored at 553 nm using a microplate reader (Thermo Fisher Scientific). The peak absorbance value was converted to molar ALA concentration using ε_553_ = 60,400 M^−1^ cm^−1^.

Inhibition of enzyme activity was determined by titrating hemin (0.1 μM–60 μM) into the substrate mixture before initiating the reaction upon enzyme addition. Hemin was dissolved in 100% dimethyl sulfoxide (DMSO) (ε_403_ nm = 170,000 M^−1^ cm^−1^) and serially diluted in 100% DMSO to prepare stock solutions for each concentration. The final DMSO concentration in the reaction mixture was 2%. All experiments were performed with a minimum of three biological replicates, each with three technical replicates. Data were fitted using nonlinear regression to generate an inhibitor dose-response curve (IC_50_) or an agonist dose-response curve (EC_50_), and statistical significance was determined using a one-way ANOVA with GraphPad Prism 10.

For inhibitor competition experiments, the apoenzymes were preincubated with hemin or PLP (0–100 μM) for 15 min at 25 °C in the dark before initiating the reaction. Next, either PLP or hemin (0–100 μM) was titrated into the reactions to measure enzyme activation after hemin inhibition or enzyme inhibition of the PLP-treated apoenzyme, respectively. For succinyl-CoA:hemin competition analyses, the WT holoenzyme was mixed with hemin (0, 25, 50, and 100 μM), followed by the addition of increasing succinyl-CoA concentrations (0, 25, 50, 100, 250, and 500 μM). All experiments were performed with a minimum of three biological replicates, each with three technical replicates. Data were fit using nonlinear regression to generate an agonist dose-response curve (EC_50_) or an inhibitor dose-response curve (IC_50_). Lineweaver-Burk analysis was performed using the resulting kinetic parameters (V_max_ and K_M_) in GraphPad Prism 10.

### In silico modeling

The mature ALAS2-heme model was generated using AlphaFold3 v3.0.0. Two copies of mature ALAS2 (residues 54–587, UniProt ID P22557) and two copies of heme b ligand were entered as the search query. Of the five models generated, the one with the highest scores was selected to generate the heme-bound model. Structure figures were generated using ChimeraX.

## Data availability

All data supporting this study are included within the article and Supplementary Information. Additional raw data are available from the corresponding author upon request.

## Supporting information

The supplemental material contains four figures and one table, including the entire ALAS enzyme multiple sequence alignment, AlphaFold models and corresponding pLDDT scores, Lineweaver-Burk plots for HRM variants, and primer sequences used for site-directed mutagenesis.

## Conflict of interest

The authors declare that they have no conflicts of interest with the contents of this article.
