# Supplementary material for: A reversible feedback mechanism regulating mitochondrial heme synthesis
Source: J Biol Chem. 2025 Dec 22;302(2):111089. doi: 10.1016/j.jbc.2025.111089 (PMC12856317; doi:10.1016/j.jbc.2025.111089)
Supplement: Supporting Figure and Tables [file mmc1.docx]

**A reversible feedback mechanism regulating mitochondrial heme synthesis**

Iva Chitrakar^1^, Alexis B. Roberson^1,2^, Pedro H. Ayres-Galhardo^1^, and Breann L. Brown^1,3*^

^1^Department of Biochemistry, Vanderbilt University School of Medicine, Nashville, Tennessee, USA, ^2^Department of Chemistry, Fisk University, Nashville, Tennessee, USA, ^3^Center for Structural Biology, Vanderbilt University School of Medicine, Nashville, Tennessee, USA

*For correspondence:

Breann L. Brown, Ph.D.

2215 Garland Ave

Nashville, TN 37232

615-343-1632

breann.brown@vanderbilt.edu

Supplementary material includes two figures and one table.


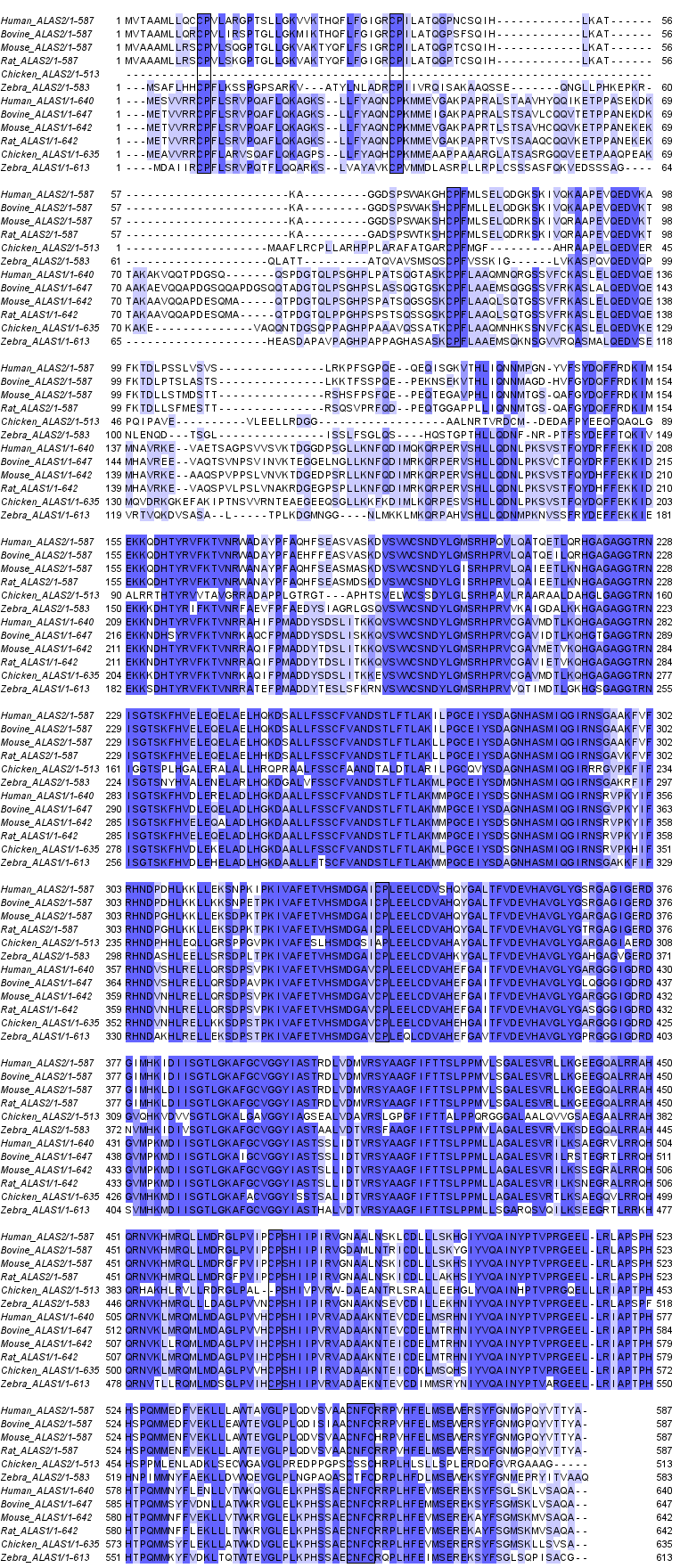


**1**

**3**

**2**

**6**

**5**


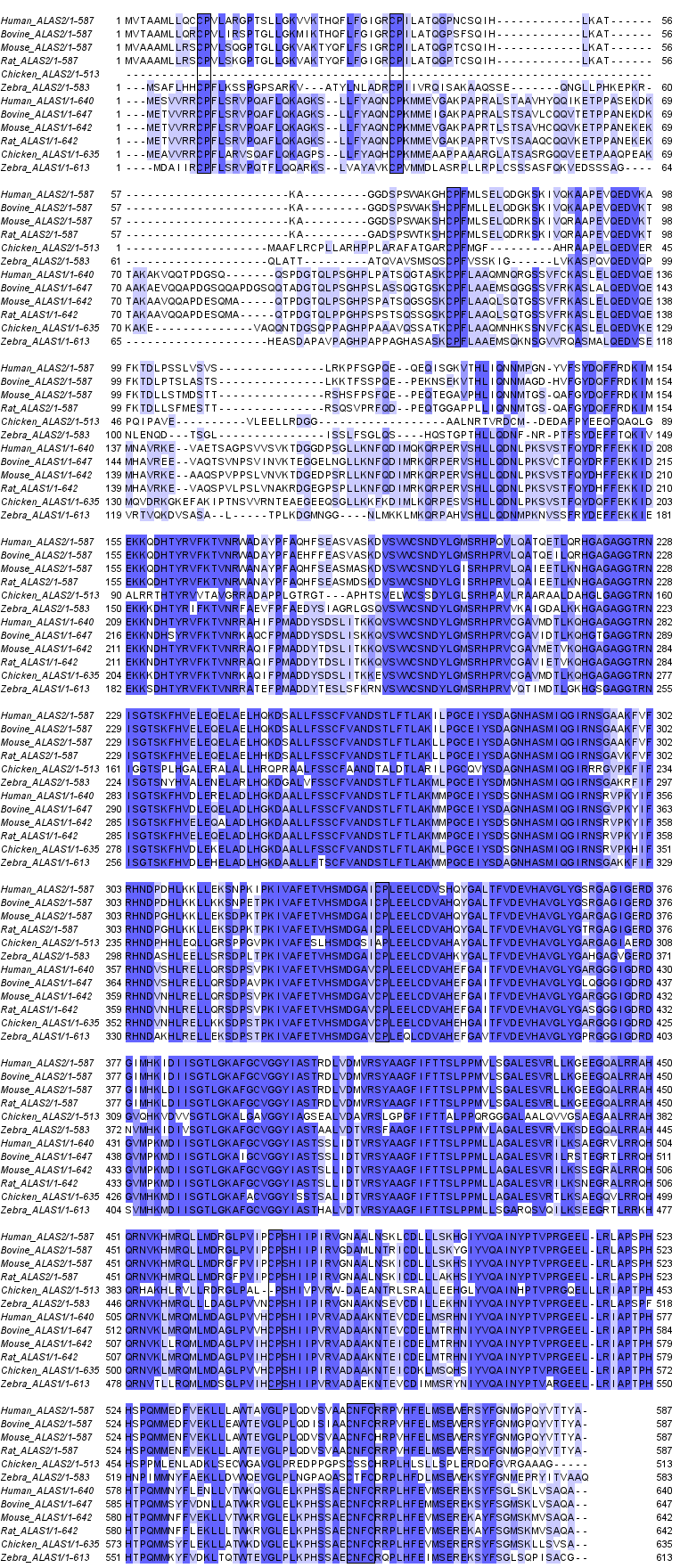


**4**

**5**

**Supplemental Figure 1.** Sequence alignment of ALAS homologs from various vertebrate organisms. Identical residues are shaded in dark blue, similar residues are shaded in light blue, and the heme regulatory motifs (HRMs) are highlighted in black boxes and numbered from N- to C-terminus.


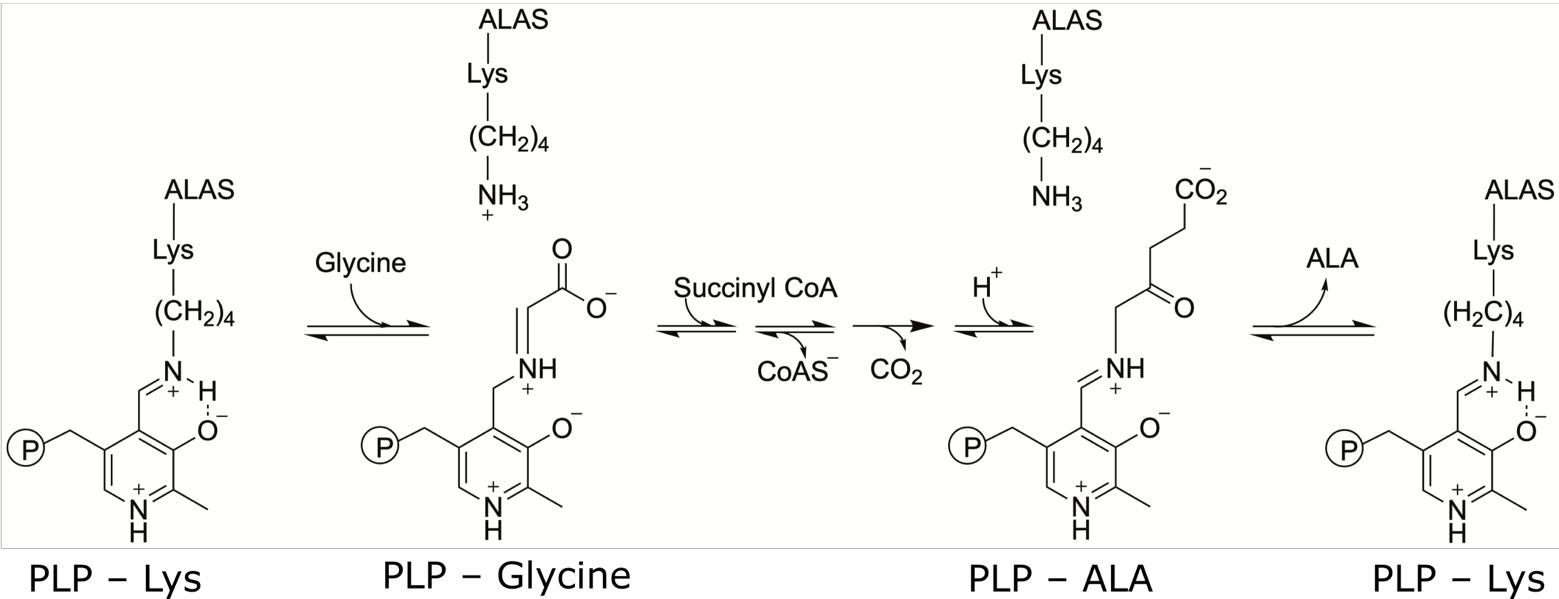


**Supplemental Figure 2.** PLP-dependent catalytic mechanism of ALAS. PLP initially forms an internal aldimine by covalently binding a conserved lysine residue in ALAS (PLP-Lys, Lys 391 for human ALAS2). Upon glycine binding, the covalent pyridoxal lysine bond is broken, and the PLP is converted to an external aldimine (PLP-Glycine). Reaction with succinyl-CoA yields an intermediate that undergoes decarboxylation and condensation to form 5-aminolevulinic acid (ALA), which is released to regenerate the internal aldimine.


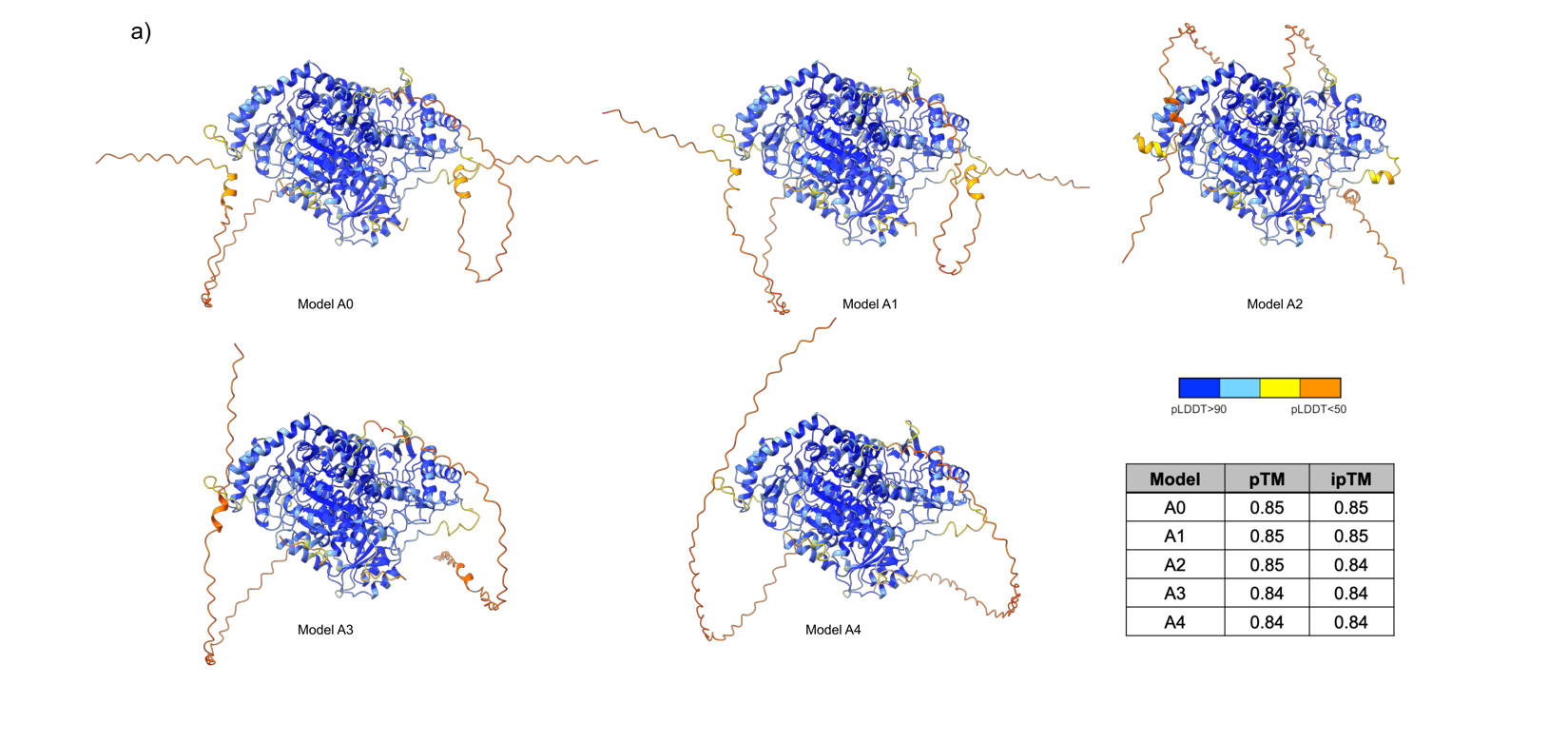


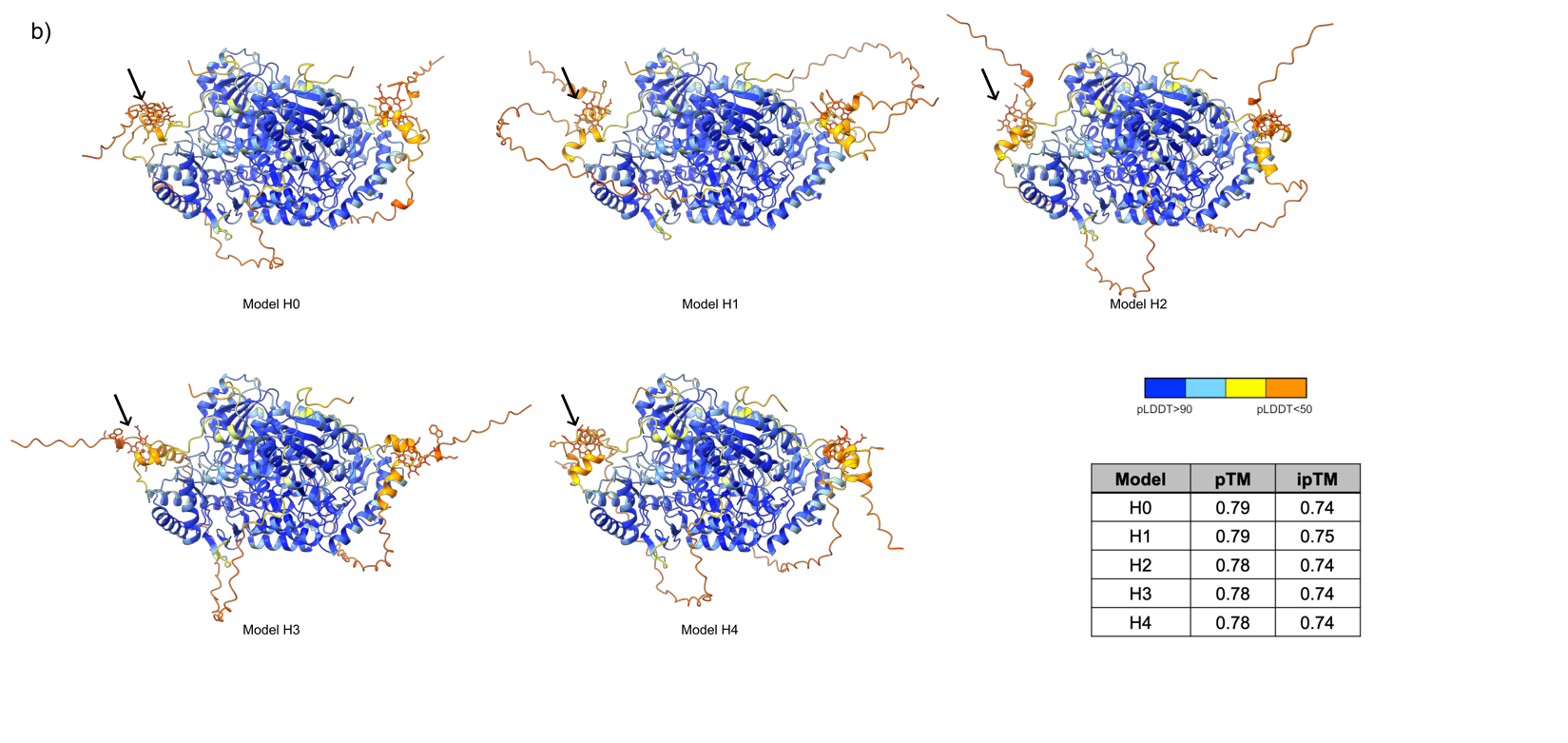


**Supplemental Figure 3.** AlphaFold3 models and associated confidence scores. The top five models output from AlphaFold3 modeling of (a) two mature human ALAS2 chains (residues 54-587) alone, and (b) two mature hALAS2 chains with two heme b molecules (stick representation, black arrows). The models are colored according to pLDDT score, where higher values represent higher model confidence. In all models, the heme molecule is docked between HRM3 (^70^CP^71^) and HRM6 (^555^CNFC^558^). The predicted template modeling (pTM) score and the interface predicted template modeling (ipTM) score are shown in the inset table.


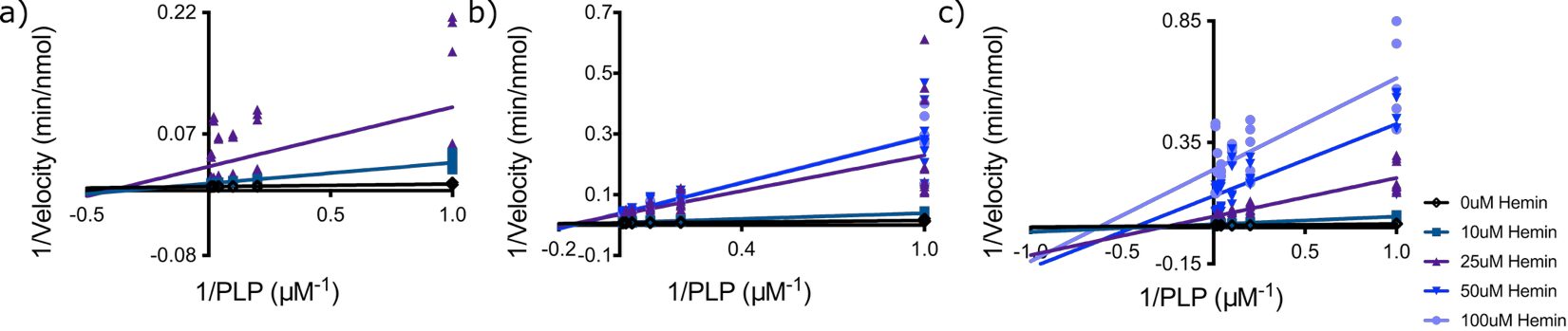


**Supplemental Figure 4.** Lineweaver Burk plots of ALAS2 a) ΔN, b) ΔC and c) Cys-mut depicting the change in reciprocal enzyme velocity with increasing concentrations of the hemin inhibitor (shades of blue).

**Supplemental Table 1. Kinetic parameters derived from Lineweaver-Burk analysis of WT ALAS2_54-587_**

| **Ligand** | **Hemin (μM)** | **K_M_ (μM)^a^** | **V_max_ (nmol/min/mg)** |
| --- | --- | --- | --- |
| PLP | 0 | 0.86 [0.58, 1.18] | 110.84 [123.30, 100.65] |
|  | 10 | 3.36 [3.05, 3.72] | 91.16 [97.56, 85.54] |
|  | 25 | 4.78 [4.02, 5.77] | 58.17 [67.25, 51.28] |
|  | 50 | 11.88 [7.19, 27.33] | 45.96 [99.50, 29.88] |
|  | 100 | 9.82 [6.93, 15.63] | 27.29 [41.39, 20.36] |
| Succinyl-CoA | 0 | 178.32 [135.81, 247.83] | 337.15 [445.63, 271.22] |
|  | 25 | 54.82 [40.02, 75.59] | 109.04 [132.82, 92.51] |
|  | 50 | 46.21 [26.96, 78.55] | 45.98 [63.13, 36.15] |
|  | 100 | 39.18 [15.68, 88.81] | 37.50 [60.98, 27.07] |

^a^95% confidence interval in brackets.

**Supplemental Table 2. Primer sequences for generating human ALAS2 constructs**

| **Construct** | **Primer sequence (5'→3')** |
| --- | --- |
| hALAS2_54-587_ (WT) | Forward: CAGACCGGTGGTAAGGCAACAAAGGCTGGA  Reverse: GTCACCACCTATGCCTGAGCGGCCGCAGGACC |
| hALAS2_75-587_ (ΔN) | Forward: AGGACCACCGGTGGCTCGGAACTCCAGGATGG  Reverse: GTCACCACCTATGCCTGAGCGGCCGCAGGACC |
| hALAS2_54-547_ (ΔC) | Forward: CAGACCGGTGGTAAGGCAACAAAGGCTGGA  Reverse: GGTGGGGCTGCCCCTCTAAGCGGCCGCAGGACC |
| hALAS2_54-587_ C(Cys-mut) | Forward (C70A): GCGAAGGGCCACGCTCCCTTCATGCTG  Reverse (C70A): CAGCATGAAGGGAGCGTGGCCCTTCGC  Forward (C555A/C558A): TGTGGCTGCCGCGAATTTCGCGCGCCGTCCTG  Reverse (C555A/C558A): GCAGCCACAGACACATCCTGG |
